# Associations of negative life events and coping styles with sleep quality among Chinese adolescents: a cross-sectional study

**DOI:** 10.1186/s12199-021-01007-2

**Published:** 2021-09-04

**Authors:** Zheng Ren, Xiumin Zhang, Yue Shen, Xiangrong Li, Minfu He, Hong Shi, Hanfang Zhao, Shuang Zha, Shuyin Qiao, Yuyu Li, Yajiao Pu, Xinwen Fan, Xia Guo, Hongjian Liu

**Affiliations:** 1 Department of Social Medicine and Health Management, School of Public Health, Jilin University, Changchun, China; 2 Department of Party and Mass Personnel, China Population Communication Center, Beijing, China; 3 Department of Epidemiology and Biostatistics, School of Public Health, Jilin University, Changchun, China

**Keywords:** Negative life events, Coping styles, Sleep quality, Adolescents

## Abstract

**Background:**

Limited published research has examined the relationships of negative life events and coping styles with sleep quality in Chinese junior high school students. We aimed to investigate the prevalence of poor sleep quality and to clarify the role of coping styles between negative life events and sleep quality.

**Methods:**

A cross-sectional study of 3081 students was conducted in Ganzhou City, Jiangxi Province, Southeastern China. Adolescent Self-Rating Life Events Checklist, Simplified Coping Style Questionnaire, and Pittsburg Sleep Quality Index were applied to assess negative life events, coping styles, and sleep quality, respectively. Descriptive analyses, independent-samples *t* tests, one-way analyses of variance, Pearson correlation analyses, and structural equation modeling (SEM) were applied to analyze the data.

**Results:**

The prevalence of poor sleep quality was 26.7%. Negative life events (*B* = 0.038, *P* < 0.001) and negative coping style (*B* = 0.049, *P* < 0.001) demonstrated a positive association with poor sleep quality, while positive coping style indicated a negative association with poor sleep quality (*B* = −0.029, *P* < 0.001). Interactions of negative life events and coping styles with sleep quality were not found (all *P* > 0.05). The association between negative life events and sleep quality was mediated by negative coping styles.

**Conclusions:**

Our results indicated that poor sleep quality was common in these Chinese adolescents. Negative life events and negative coping style were associated with an increased prevalence of poor sleep quality, while the positive coping style was related to a decreased prevalence of poor sleep quality. A negative coping style mediated the association between negative life events and sleep quality.

**Supplementary Information:**

The online version contains supplementary material available at 10.1186/s12199-021-01007-2.

## Background

Adolescence is a crucial transitional period from childhood to adulthood and is accompanied by increasing stress, significant social readjustments, and high reactivity to stress [1]. Sleep quality is one of the most important indices of adolescent health because sleep has a significant influence on the development of psychophysiological functions, including attention, emotion, and behavior [2–4]. Sleep problems are prevalent in adolescents. A review and meta-analysis of worldwide sleep patterns and problems during adolescence showed that insomnia and daytime sleepiness were two main types of sleep problems [5]. Studies in Western countries have estimated that 35%-56% of adolescents have poor sleep quality [6–8]. A previous study conducted in China revealed that 20.0% of the sample was troubled with poor sleep quality [9]. Adolescents’ sleep problems have been a major international public health problem. Poor sleep quality brings all kinds of negative consequences, including impairments of adolescents’ learning capacity, school performance, psychosocial health, and neurobehavioral functioning, and can cause diseases [10–12].

There are many factors experienced during adolescence that may cause poor sleep. The period of adolescence is characterized by the rapid development of psychology, sociology, and biology [13], each of which has the potential to promote stress. Stress in the form of negative life events is common in adolescence. Negative life events refer to things that compel people to make changes in their ongoing life patterns [14]. Interpersonal relationships (family relationship, teacher-student relationship, peer relationship), academic pressure, death of a relative, and having something valuable lost or stolen are all included in the category of negative life events. Previous studies have reported that a variety of negative life events are associated with sleep problems [15, 16]. It has been found that the presence of negative life events is a risk factor for poor sleep quality [17]. Proposed by Lanzarus, coping process theory [18] holds that coping is a process of ongoing cognitive and behavioral efforts to manage specific external and/or internal demands that are assessed as consuming or exceeding personal resources, and it adapts to pressure by evaluating stressors and applying coping strategies. Generally, coping styles include two categories: a positive coping style of seeking help to solve problems or optimistically facing stressful situations and a negative coping style of dealing with stress through fantasy denial or unhealthy behavior. Coping styles are an individual’s unique inertia behavior, which will make the individual’s psychology and physiology change in the face of life events, and further affect the sleep quality. Coping styles, as cognitive and behavioral strategies employed in response to the appraisal of stress, are essential to consider in the context of adolescent stress and sleep [19]. Previous studies have researched the relationships among negative life events, coping strategies, and sleep quality in patients [20, 21] or young adults [22], and few studies have examined the relationships in school adolescents, especially junior high school students, who spend most of their time in school. These students are immature in mind and poor in social cognitive ability, but school stressors are difficult to avoid [23], and it is very important to understand the relationships among these three factors for improving students’ sleep problems.

Therefore, the aims of the current study were to (1) investigate the prevalence of poor sleep quality in a sample of Chinese adolescents aged 11 to 16 years, (2) explore the associations of negative life events and coping styles with sleep quality, and (3) clarify the role of coping styles between negative life events and sleep quality.

## Methods

### Study design and participants

The data used were obtained from a cross-sectional study conducted in Ganzhou City, Jiangxi Province, Southeast China, from September to October 2017. Four regions were selected for this study: two in an urban setting and two in a rural setting. We chose two junior high schools at each survey point. In total, eight schools were selected, and each school had 3 grades (grade 7 to grade 9). Three classes were randomly selected from each grade in each school. There were 72 classes in the selected schools included in the study. The participants completed questionnaires in the classroom during regular class time, with a research team member in attendance. There were 3176 questionnaires were collected from participants. After deleting the questionnaires with missing data, this study collected a total of 3081 valid questionnaires, and the valid response rate was 97.0%. This study received approval from the Ethics Committee of Jilin University School of Public Health (No. 2017-08-16). Written informed consent was obtained from the participants and their parents or legal guardians before their participation in the survey.

### Sample information

The information was obtained using one self-administered questionnaire created by the research team. We collected sociodemographic variables, lifestyle variables, and self-rated health variables as follows: gender, grade, school type, family type, parental education level, smoking, drinking alcohol, physical exercise, self-rated health, self-perceived study stress, and depressive symptoms. Regarding smoking and drinking alcohol, participants were classified as nonsmokers/nondrinkers if they had never smoked or drunk and as smokers/drinkers if they had experimented or if they smoked/drank regardless of the frequency and quantity [24]. The Chinese Secondary School Students Depression Scale (CSSSDS) was used to measure the depressive symptoms of participants [25]. The scale includes 20 items, and each item has a rating ranging from 1 to 5 points. The total average depressive symptoms scores were calculated by the sum of the scores of all items and divided by 20. A participant who had a total average score of at least 2 was defined as having depressive symptoms [26]. Cronbach’s alpha of the CSSSDS was 0.94 in the present study.

### Adolescent Self-Rating Life Events Checklist (ASLEC)

Negative life events were assessed by the Adolescent Self-Rating Life Events Checklist (ASLEC), which evaluated whether such events had occurred and the impact of the negative life events experienced in the past 12 months [27]. The ASLEC consisted of 27 items of negative life events, including 6 subscales interpersonal relationships, academic pressure, being punished, loss, change for adaptation, and others (Supplementary Material). Each item is rated on a 6-point Likert-type scale. If participants answered “no,” the score was 0 (not occur); when participants answered “yes,” they were required to assess the impact of the negative life event from 1 (no impact at all) to 5 (very strong impact). High scores show that negative life events have a more serious impact on individuals. Cronbach’s alpha of the ASLEC was 0.93 in this study.

### Simplified Coping Style Questionnaire (SCSQ)

The Simplified Coping Style Questionnaire (SCSQ) adapted by a Chinese scholar [28] contained 20 items, and it was divided into two coping dimensions: positive coping styles including items 1–12, and negative coping styles including items 13–20 (Supplementary Material). The individuals evaluated their typical coping attitude and methodology on a 4-point Likert scale (never, sometimes, often, and always). For each of the two coping styles, high scores indicate the specific coping styles that individuals often use when dealing with problems. Cronbach’s alphas were 0.89 for positive coping style and 0.74 for negative coping style in the current study.

### Pittsburgh sleep quality index (PSQI)

Sleep quality was assessed with the Pittsburg Sleep Quality Index (PSQI), an 18-item scale that evaluated seven components of sleep quality in the most recent month, including subjective sleep quality, sleep latency, sleep duration, sleep efficiency, sleep disturbances, sleep medication use, and daytime dysfunction [29]. Each component is assessed on a 4-point Likert scale with a range in global score from 0 to 21, with higher scores indicating poorer sleep quality. A PSQI global score > 5 yields a diagnostic sensitivity of 89.6% and specificity of 86.5% in distinguishing good and poor sleepers [29]. Therefore, a total score less than or equal to 5 indicates “good sleep quality,” while a score above 5 indicates “poor sleep quality” in this study. Cronbach’s alpha of the PSQI with the study sample was 0.71.

### Data analysis

Descriptive statistics were used to examine the sample demographic characteristics of the participants, and the data were presented as the mean with standard deviation (SD) and number (proportions). The study variables were compared among grade groups, family type groups, fathers’ education level groups, mothers’ education level groups, self-rated health groups, and self-perceived study stress groups via one-way analysis of variance (ANOVA). *T* tests were performed to examine the differences in gender, school type, smoking, drinking alcohol, physical exercise, and depressive symptoms groups. Univariate logistic regression analyses were used to analyze the associations between poor sleep quality and selected factors. The odds ratios (ORs) were calculated as the exponentiated coefficient from logistic models. Pearson correlation analysis was used to analyze the correlation among the variables of negative life events, coping styles, and sleep quality. Multivariable logistic regression analyses were used to examine associations between variables (negative life events, coping styles, and negative life events × coping styles) and poor sleep quality. There were differences between participants with a positive coping style and participants with a negative coping style if the coefficients of the interaction terms of negative life events × coping styles were significant. Structural equation modeling (SEM) was employed to further test the relationships among the variables of negative life events, coping styles, and sleep quality. The SEM used bootstrap maximum likelihood estimation. All *p* values were two-sided, and values less than 0.05 were considered statistically significant. These analyses were performed with SPSS 24.0 and AMOS 23.0 (IBM Corp, Armonk, New York, USA).

## Results

### Associations of sample characteristics with negative life events and coping styles

The demographics of the participants and distributions of negative life events and coping styles in categorical items are shown in Table 1. The study sample consisted of 3081 middle school students with an average age of 13.5 ± 1.1 years. The numbers of participants in grades 7, 8, and 9 were 979 (31.8%), 1085 (35.2%), and 1017 (33.0%), respectively. Mean negative life events scores differed across the distributions of grade, school type, family type, parents’ education level, smoking, drinking alcohol, self-rated health, self-perceived study stress, and depressive symptoms groups (*P* < 0.001). Mean positive coping style score differed across the distributions of all sample characteristics except depressive symptoms groups. Mean negative coping style score differed across the distributions of grade, mothers’ education level, smoking, drinking alcohol, self-rated health, self-perceived study stress, and depressive symptoms groups (*P* < 0.05).

**Table 1:** Sample characteristics and the distributions of negative life events and coping styles in categorical items (*N*=3081)

Variables	Total *n* (%)	Negative life events		Positive coping style		Negative coping style	
		M (SD)	*P*	M (SD)	*P*	M (SD)	*P*
Gender			0.113		<0.001		0.064
Male	1565 (50.8)	52.7 (20.2)		15.4 (8.3)		6.5 (4.5)	
Female	1516 (49.2)	51.6 (18.5)		16.6 (7.9)		6.8 (4.3)	
Grades			<0.001		<0.001		<0.001
7	979 (31.8)	46.7 (17.1)		14.2 (8.4)		5.8 (4.4)	
8	1085 (35.2)	53.3 (19.7)		15.8 (7.9)		6.5 (4.3)	
9	1017 (33.0)	56.2 (19.8)		17.9 (7.7)		7.7 (4.4)	
School type			<0.001		<0.001		0.704
Urban	1552 (50.4)	50.4 (18.6)		17.0 (8.1)		6.6 (4.4)	
Rural	1529 (49.6)	54.0 (19.9)		14.9 (8.0)		6.7 (4.5)	
Family type			<0.001		0.042		0.662
Stem family	1060 (34.4)	52.6 (19.5)		16.4 (8.1)		6.7 (4.4)	
Nuclear family	1669 (54.2)	51.0 (18.9)		15.9 (8.3)		6.6 (4.5)	
Single parent family	271 (8.8)	56.4 (20.6)		15.1 (7.8)		6.8 (4.4)	
Foster family	81 (2.6)	57.3 (19.8)		15.0 (7.1)		7.0 (4.0)	
Fathers’ education level			<0.001		<0.001		0.342
Junior college or higher	334 (10.8)	48.0 (18.6)		18.2 (8.7)		6.4 (4.5)	
Senior school	671 (21.8)	50.9 (18.9)		16.8 (7.4)		6.8 (4.5)	
Junior middle school	1647 (53.5)	52.2 (18.8)		15.4 (8.2)		6.6 (4.4)	
Primary school or less	429 (13.9)	57.2(21.6)		15.1(8.0)		7.0(4.5)	
Mothers’ education level			<0.001		<0.001		0.015
Junior college or higher	252 (8.2)	48.5 (19.2)		18.3 (8.7)		6.8 (4.6)	
Senior school	464 (15.1)	51.5 (20.3)		17.4 (8.3)		6.8 (4.6)	
Junior middle school	1447 (46.9)	50.5 (17.8)		15.6 (8.1)		6.4 (4.3)	
Primary school or less	918 (29.8)	56.1 (20.6)		15.1 (7.8)		7.0 (4.5)	
Smoking			<0.001		0.015		0.004
No	3003 (97.5)	51.7 (18.8)		16.0 (8.1)		6.6 (4.4)	
Yes	78 (2.5)	71.1 (28.4)		13.8 (8.1)		8.6 (5.7)	
Drinking alcohol			<0.001		0.010		<0.001
No	2086 (67.7)	49.4 (17.7)		16.2 (8.3)		6.4 (4.4)	
Yes	995 (32.3)	57.9 (21.3)		15.4 (7.8)		7.3 (4.5)	
Physical exercise			0.050		<0.001		0.208
Lack of exercise	2251 (73.1)	52.6 (19.0)		15.0 (7.8)		6.6 (4.3)	
Often exercise	830 (26.9)	51.0 (20.1)		18.5 (8.5)		6.8 (4.7)	
Self-rated health			<0.001		<0.001		<0.001
Good	1724 (56.0)	49.4 (18.5)		16.7 (8.3)		6.5 (4.5)	
Fair	1168 (37.9)	54.4 (18.8)		14.9 (7.8)		6.7 (4.3)	
Poor	189 (6.1)	63.5 (23.8)		15.5 (8.0)		8.2 (4.9)	
Self-perceived study stress			<0.001		0.020		<0.001
Low	536 (17.4)	42.8 (15.7)		16.8 (8.9)		5.9 (4.3)	
Fair	1554 (50.4)	49.9 (17.1)		15.6 (8.0)		6.3 (4.3)	
High	991 (32.2)	60.9 (20.9)		16.0 (7.9)		7.6 (4.6)	
Depressive symptoms			<0.001		0.705		<0.001
No	2467 (80.1)	46.8 (14.8)		15.9 (8.4)		6.0 (4.1)	
Yes	614 (19.9)	73.5 (20.6)		16.1 (7.0)		9.4 (4.6)	

Note: *M(SD)* mean and standard deviation

Differences were assessed using *t* test for gender, school type, smoking, drinking alcohol, physical exercise and depressive symptoms, and ANOVA for other variables

### Associations between poor sleep quality and selected factors

The prevalence of poor sleep quality was 26.7% (822/3081). Compared with participants in grade one, participants in grade two and grade three were 1.588 (OR = 1.588, 95% CI = [1.289, 1.956]) and 2.194 (OR = 2.194, 95% CI = [1.785, 2.696]) times as likely to have poor sleep quality, respectively. The risk of poor sleep quality of participants studying in rural schools was 1.453 (OR = 1.453, 95% CI = [1.237, 1.706]) times that of participants studying in urban schools. A higher negative life events score and negative coping style score were associated with a higher risk of poor sleep quality (OR = 1.049, 95% CI = [1.044, 1.054]; OR = 1.099, 95% CI = [1.080, 1.119], respectively). Details of the results of the cross-sectional analysis of poor sleep quality in participants with different characteristics are described in Table 2.

**Table 2:** Univariate logistic regression analyses of poor sleep quality (*N*=3081)

Variables	Poor sleep quality		*P*	OR (95%CI)
	No (*n*=2259)	Yes (*n*=822)		
Genders^a^				
Male	1167 (74.6)	398 (25.4)	–	1.000
Female	1092 (72.0)	424 (28.0)	0.112	1.138 (0.970, 1.336)
Grades^a^				
First	794 (81.1)	185 (18.9)	–	1.000
Second	792 (73.0)	293 (27.0)	<0.001	1.588 (1.289, 1.956)
Third	673 (66.2)	344 (33.8)	<0.001	2.194 (1.785, 2.696)
School types^a^				
Urban	1194 (76.9)	358 (23.1)	–	1.000
Rural	1065 (69.7)	464 (30.3)	<0.001	1.453 (1.237, 1.706)
Family types^a^				
Stem family	777 (73.3)	283 (26.7)	–	1.000
Nuclear family	1259 (75.4)	410 (24.6)	0.212	0.894 (0.750, 1.066)
Single-parent family	172 (63.5)	99 (36.5)	0.001	1.580 (1.192, 2.096)
Foster family	51 (63.0)	30 (37.0)	0.046	1.615 (1.008, 2.587)
Fathers’ education levels^a^				
Junior college or higher	266 (79.6)	68 (20.4)	–	1.000
Senior school	511 (76.2)	160 (23.8)	0.214	1.225 (0.889, 1.687)
Junior middle school	1201 (72.9)	446 (27.1)	0.011	1.453 (1.090, 1.937)
Primary school or less	281 (65.5)	148 (34.5)	<0.001	2.060 (1.477, 2.873)
Mothers’ education levels^a^				
Junior college or higher	196 (77.8)	56 (22.2)	–	1.000
Senior school	352 (75.9)	112 (24.1)	0.564	1.114 (0.773, 1.605)
Junior middle school	1083 (74.8)	364 (25.2)	0.320	1.176 (0.854, 1.620)
Primary school or less	628 (68.4)	290 (31.6)	0.004	1.616 (1.164, 2.244)
Smoking^a^				
No	2226 (74.1)	777 (25.9)	–	1.000
Yes	33 (42.3)	45 (57.7)	<0.001	3.907 (2.475, 6.167)
Drinking alcohol^a^				
No	1623 (77.8)	463 (22.2)	–	1.000
Yes	636 (63.9)	359 (36.1)	<0.001	1.979 (1.677, 2.335)
Physical exercises^a^				
Often exercise	628 (75.7)	202 (24.3)	–	1.000
Lack of exercise	1631 (72.5)	620 (27.5)	0.074	1.182 (0.984, 1.420)
Self-rated health^a^				
Good	1381 (80.1)	343 (19.9)	–	1.000
Fair	790 (67.6)	378 (32.4)	<0.001	1.926 (1.625, 2.284)
Poor	88 (46.6)	101 (53.4)	<0.001	4.621 (3.392, 6.296)
Self-perceived study stresses^a^				
Low	478 (89.2)	58 (10.8)	–	1.000
Fair	1211 (77.9)	343 (22.1)	<0.001	2.334 (1.733, .144)
High	570 (57.5)	421 (42.5)	<0.001	6.087 (4.508, 8.218)
Depressive symptoms^a^				
No	2019 (81.8)	448 (18.2)	–	1.000
Yes	240 (39.1)	374 (60.9)	<0.001	7.023 (5.798, 8.507)
Negative life events^b^	47.5 (16.2)	65.0 (21.2)	<0.001	1.049 (1.044, 1.054)
Interpersonal relationships	10.1 (4.4)	14.1 (4.9)	<0.001	1.190 (1.168, 1.212)
Academic pressure	10.4 (4.0)	14.0 (4.8)	<0.001	1.206 (1.182, 1.230)
Being punished	11.1 (4.6)	15.2 (6.3)	<0.001	1.139 (1.122, 1.157)
Loss	4.6 (2.6)	6.3 (3.6)	<0.001	1.178 (1.149, 1.209)
Change for adaptation	5.6 (2.1)	7.6 (3.2)	<0.001	1.351 (1.305, 1.398)
Others	5.6 (2.2)	7.8 (3.6)	<0.001	1.306 (1.266, 1.347)
Coping styles^b^				
Positive coping style	16.0 (8.3)	15.8 (7.6)	0.566	0.997 (0.987, 1.007)
Negative coping style	6.2 (4.2)	8.1 (4.7)	<0.001	1.099 (1.080, 1.119)

Note:

^a^Categorical variables are presented as the frequencies and percentages

^b^Continuous variables are presented as the mean and standard deviation

Poor sleep quality was assessed with the PSQI

#### Analysis of correlations

The means, standard deviations, and bivariate correlations for negative life events, coping styles, and sleep quality are presented in Table 3. Negative life events were positively correlated with positive coping style (*r* = 0.115, *P* < 0.001) and negative coping style (*r* = 0.340, *P* < 0.001). Negative life events (*r* = 0.506, *P* < 0.001) and negative coping style were positively correlated with sleep quality (*r* = 0.245, *P* < 0.001). Positive coping style was not correlated with sleep quality.

**Table 3:** Descriptive statistics and correlation among variables

Variables	1	2	3	4
1. Negative life events	1			
2. Positive coping style	0.115^***^	1		
3. Negative coping style	0.340^***^	0.423^***^	1	
4. Sleep quality	0.506^***^	−0.026	0.245^***^	1
M	52.2	16.0	6.7	4.3
SD	19.3	8.1	4.4	2.6

Note:

^***^P < 0.001

#### The associations of negative life events and coping styles with poor sleep quality

Table 4 presents the associations of negative life events and coping styles with poor sleep quality. Model 1 with only the main effect showed that for the participants, negative life events and negative coping style presented a positive association with poor sleep quality, indicating that participants with higher scores of negative life events and negative coping style were more likely to experience poor sleep quality (*B* = 0.046, *P* < 0.001; *B* = 0.065, *P* < 0.001, respectively). The participants with higher positive coping style scores were more likely to have a lower prevalence of poor sleep quality (*B* = −0.036, *P* < 0.001). The analysis of adjusted model 2 and model 3 also demonstrated the same results as model 1. Whether adjusted or not, interactions of negative life events and coping styles with poor sleep quality were not found (all *P* > 0.05).

**Table 4:** Multivariable logistic regression analysis of negative life events and coping styles on poor sleep quality

Variables	Model 1		Model 2		Model 3	
	B	*P*	B	*P*	B	*P*
Model with only main effect						
Negative life events	0.046	<0.001	0.045	<0.001	0.029	<0.001
Positive coping style	−0.036	<0.001	−0.041	<0.001	**-**0.021	0.003
Negative coping style	0.065	<0.001	0.062	<0.001	0.029	0.020
Model with interactions						
Negative life events	0.054	<0.001	0.052	<0.001	0.035	<0.001
Positive coping style	−0.033	0.111	−0.039	0.062	**-**0.032	0.131
Negative coping style	0.110	0.001	0.100	0.003	0.090	0.009
Negative life events and Positive coping style	0.000	0.803	0.000	0.852	0.000	0.633
Negative life events and Negative coping style	−0.001	0.143	−0.001	0.222	**-**0.001	0.057

Note:

Model 1: Unadjusted

Model 2: Adjusted for gender and grade

Model 3: Adjusted for gender, grade, school type, family type, fathers’ education level, mothers’ education level, smoking, drinking alcohol, physical exercise, self-rated health, self-perceived study stress, and depressive symptoms

*P* was calculated by analysis of multivariable logistic regression

**Fig. 1:**
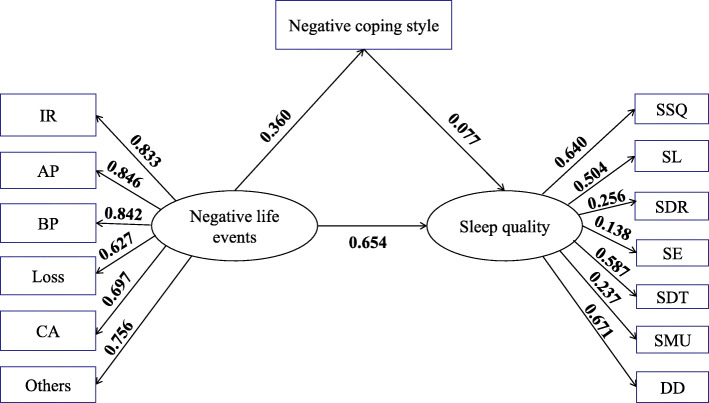
Standardized estimation of the associations of negative life events and negative coping style with sleep quality. Note: IR interpersonal relationships, AP academic pressure, BP being punished, CA change for adaptation, SSQ subjective sleep quality, SL sleep latency, SDR sleep duration, SE sleep efficiency, SDT sleep disturbance, SMU sleep medication use, DD daytime dysfunction

On the basis of the multivariable logistic regression analysis, we further constructed a structural equation model for the associations of negative life events and coping styles with sleep quality. There was no significant correlation between positive coping style and sleep quality in the correlation analysis; thus, they were not included in the path model. Figure 1 shows the SEM for the associations of negative life events and negative coping style with sleep quality. The model had an acceptable fit (*χ*^*2*^ = 642.763, RMSEA = 0.052, NFI = 0.962, TLI = 0.955, PNFI = 0.729, CFI = 0.966), and there were significant differences in all paths in the structure (all *P* < 0.001).

Table 5 presents the direct, indirect, and total effects of negative life events and negative coping style with sleep quality. As shown, negative life events had both direct (0.654) and indirect effects (0.028) on sleep quality, while negative coping style had only a direct effect (0.077). Among all the dimensions of negative life events and sleep quality, the absolute values of academic pressure (0.846) and daytime dysfunction (0.671) were the highest.

**Table 5:** Standardized effects on sleep quality from the SEM analysis

Variables	Direct effect	Indirect effect	Total effect
Negative life events	0.654	0.028	0.682
Negative coping style	0.077	0.000	0.077

## Discussion

The present study focused on the status of sleep quality and the associations of negative life events and coping styles with sleep quality among Chinese adolescents. We found that (1) poor sleep quality was not rare among these Chinese adolescents, with a prevalence of 26.7%, (2) negative life events and negative coping style were associated with an increased prevalence of poor sleep quality, (3) positive coping style was related to a decreased prevalence of poor sleep quality, and (4) negative coping style mediated the association between negative life events and sleep quality. Our findings provide valuable information for developing sleep guidance for adolescents, and those involved in public health education should encourage adolescents to establish healthy sleep patterns.

Using the PSQI with a cut-off score of 5, this study found that the prevalence of poor sleep quality was 26.7% among Chinese adolescents. Studies of South Indian [30], Turkish [31], Lebanese [32], and Swedish [33] adolescents showed that the prevalence of poor sleep quality was 2.5%, 36.4%, 58.7%, and 76%, respectively, with great differences. Evidence from an epidemiological study of sleep quality in adolescents in southern China [34] found that the prevalence of poor sleep quality was 34.32%. The difference in prevalence might be related to the samples and regions. The participants of our study are junior high school students distributed in rural and urban areas, while the study in South India included only adolescents in rural areas, and rural residence may lead to a low prevalence among participants; another study in China includes not only junior high school students (grades 7–9) but also senior high school students (grades 10–12), and students in the high school stage may suffer from greater study stress, resulting in a high prevalence of poor sleep quality. Our research results deserve the attention of parents and school teachers, and measures should be taken to improve students’ sleep quality.

After adjusting for potential confounding factors including sociodemographic variables, lifestyle variables, and self-rated health variables, negative life events were found to be a significant influencing factor of poor sleep quality in the current study. These findings are in line with those of studies identifying that stressful life events are related to poor sleep quality [35, 36]. As a stressor, negative life events are various, and their effects may be presented through a maladaptive hypothalamic-pituitary-adrenal (HPA) system [37]. From the perspective of psychophysiology, stress caused by negative life events may lead to greater activation of the locus coeruleus norepinephrine system and HPA axis, which can increase excitement and aggravate difficulty in falling asleep [38]. All kinds of stress reactions caused by negative life events lead to dysfunction of the nervous system and abnormal sleep patterns. In addition, it may be that negative life events induce negative emotions such as anxiety and sadness, which affect sleep quality.

The results of our study demonstrated that individuals who adopted more negative coping styles were more likely to suffer from poor sleep quality. Similar findings have been observed in studies among adolescents conducted in other parts of the country [39, 40]. Individuals who adopt a negative coping style may face stress through disengagement or social isolation, which tends to increase the risk of psychological problems, thus affecting the quality of sleep. In the main effect of multivariable regression analysis in our study, it was found that a positive coping style can reduce the risk of poor sleep quality. Thinking positively and solving problems usually emerged as a good and positive coping strategy. A previous study found that thinking positively is considered to improve sleep quality because it develops the ability to explore new approaches of adaptation through re-examining the cognitive process, suppressing feelings of anxiety, and seeking to divert attention [41].

In addition, we explored the interactions of negative life events and coping styles with sleep quality in participants, but no interactions were found regardless of whether other confounding factors were adjusted. In contrast, a previous study indicated that changes in sleep were significantly moderated by an individual’s coping style [42]. The reasons for this are unclear, but there may be other influencing mechanisms between variables. Based on the results from the structural equation modeling, we found that negative coping style mediated the association between negative life events and sleep quality, which is consistent with previous study findings [43]. The use of a negative coping style (such as emotion-focused coping and avoidance coping) in the face of stressful events was associated with more sleep problems [44]. When faced with negative life events, individuals’ adoption of a negative coping style triggers their own negative cognition about stressful events, which increases the risk of poor sleep quality and brings sleep distress. According to the theory of stress cognitive insomnia [45], when stressful events occur, people’s excessive worry about stressful events before falling asleep arouses their anxiety, causes cognitive bias and distorted cognitive evaluation of stressful events, and leads to the use of a negative coping style, which tends to decrease sleep quality.

Our study has several strengths. First, we investigated not only the interactions of negative life events and coping styles with sleep quality but also the mediating role of negative coping style in the association of negative life events and sleep quality in Chinese school adolescents. Second, to improve the reliability of the results, our study adjusted for most of the confounding factors, such as gender, grade, school type, family type, fathers’ education level, mothers’ education level, smoking, drinking alcohol, physical exercise, self-rated health, self-perceived study stress, and depressive symptoms.

Some limitations of our study must be acknowledged. First, a cross-sectional survey design cannot determine causal relations among study variables. Second, there may be recall bias in the information collection process because all the information in this study stemmed from self-reported questionnaires completed by the participants. Third, the participants of this study were only adolescents aged 11–16 from Ganzhou City, Jiangxi Province, China, probably limiting the generalization of the findings.

## Conclusions

Our results indicated that poor sleep quality was common in these Chinese adolescents. Negative life events and negative coping style were associated with an increased prevalence of poor sleep quality, while the positive coping style was related to a decreased prevalence of poor sleep quality. Negative coping style mediated the association between negative life events and sleep quality. Much attention should be paid to the association between negative life events and sleep quality among adolescents. Encouraging students to positively cope with stressors can help prevent sleep problems.

## Supplementary Information

**Additional file 1.**

## Data Availability

The datasets used and/or analyzed during the current study are available from the corresponding author on reasonable request.
