# Supplementary material for: Associations of negative life events and coping styles with sleep quality among Chinese adolescents: a cross-sectional study
Source: Environ Health Prev Med. 2021 Sep 4;26:85. doi: 10.1186/s12199-021-01007-2 (PMC8418725; doi:10.1186/s12199-021-01007-2)
Supplement: Supplementary file 1 — Additional file 1. [file 12199_2021_1007_MOESM1_ESM.doc]

**Adolescent Self-Rating Life Events Checklist (ASLEC)**

1. Be misunderstood by others

2. Be discriminated against

3. Failure in examinations

4. Have a dispute with classmates or friends

5. Marked changes in daily life

6. Do not enjoy school life

7. Failure in love

8. Away from family for a long time

9. Heavy learning burden

10. A tense relationship with teachers

11. Suffer from serious diseases

12. Relatives and friends suffer from serious diseases

13. Death of relatives and friends

14. Stolen or lost something

15. Lose face in public

16. Financial difficulties in family

17. Contradictions in family

18. Failure in expected selection (such as merit students)

19. Be criticized or disciplined

20. Transfer or suspend schooling

21. Be fined

22. Pressure to enter a higher school

23. Fight with others

24. Be beaten by parents

25. Study pressure from family

26. An unexpected fright or accident

27. Other events

**Simplified Coping Style Questionnaire (SCSQ)**

1. I get out of trouble through study, work, or other events.

2. I pour out my inner troubles by talking to others.

3. I try to look on the bright side of things.

4. I change my mind and rediscover what is important in life.

5. I do not take the problem too seriously.

6. I stick to my stance and fight for what I want.

7. I solve the problem by finding out several different ways.

8. I ask classmates, friends, or relatives to give advice.

9. I change some of my problems or original practices.

10. I learn from other people’s ways of dealing with similar difficult situations.

11. I pursue hobbies and play an active part in sports and cultural activities.

12. I try to control my anger, sadness, regret, and disappointment.

13. I try to put my problems aside for the moment by taking a rest or vacation.

14. I relieve my troubles by smoking, drinking, taking medicine, and eating.

15. I think waiting is the only thing to do because time changes things.

16. I try to forget the whole thing.

17. I solve the problem by depending on others.

18. I accept the reality because there is no other way.

19. I fantasize that the status quo may change when a miracle happens.

20. I comfort myself.
